# Identifying Electroencephalography Biomarkers in Individuals at Clinical High Risk for Psychosis in an International Multi-Site Study

**DOI:** 10.3389/fpsyt.2022.828376

**Published:** 2022-03-16

**Authors:** Sarah Kerins, Judith Nottage, Gonzalo Salazar de Pablo, Matthew J. Kempton, Stefania Tognin, Dorien H. Niemann, Lieuwe de Haan, Thérèse van Amelsvoort, Jun Soo Kwon, Barnaby Nelson, Romina Mizrahi, Philip McGuire, Paolo Fusar-Poli, The PSYSCAN Consortium, Philip McGuire

**Affiliations:** ^1^Department of Psychosis Studies, Institute of Psychiatry, Psychology and Neuroscience, King's College London, London, United Kingdom; ^2^Early Psychosis: Interventions and Clinical-Detection Lab, Department of Psychosis Studies, Institute of Psychiatry, Psychology and Neuroscience, King's College London, London, United Kingdom; ^3^Department of Neuroimaging, Institute of Psychiatry, Psychology and Neuroscience, King's College London, London, United Kingdom; ^4^Institute of Psychiatry and Mental Health, CIBERSAM, Madrid, Spain; ^5^Department of Child and Adolescent Psychiatry, Hospital General Universitario Gregorio Marañón School of Medicine, Instituto de Investigación Sanitaria Gregorio Marañón, Universidad Complutense, CIBERSAM, Madrid, Spain; ^6^Outreach and Support in South London (OASIS), South London and Maudsley NHS Foundation Trust, London, United Kingdom; ^7^Department of Psychiatry, University Medical Center Utrecht Brain Center, Utrecht, Netherlands; ^8^Department of Psychiatry, Early Psychosis Section, Amsterdam UMC, University of Amsterdam, Amsterdam, Netherlands; ^9^Department of Psychiatry and Neuropsychology, Maastricht University, Maastricht, Netherlands; ^10^Department of Psychiatry, Seoul National University College of Medicine, Seoul, South Korea; ^11^Orygen, The National Centre of Excellence in Youth Mental Health, Melbourne, VIC, Australia; ^12^Centre for Youth Mental Health, University of Melbourne, Melbourne, VIC, Australia; ^13^Douglas Mental Health University Institute, Montreal, QC, Canada; ^14^Department of Psychiatry, McGill University, Montreal, QC, Canada; ^15^National Institute for Health Research, Mental Health Biomedical Research Centre, South London and Maudsley NHS Foundation Trust, King's College London, London, United Kingdom; ^16^Department of Brain and Behavioral Sciences, University of Pavia, Pavia, Italy

**Keywords:** EEG, CHR-P, multi-site, biomarkers, psychosis prediction

## Abstract

**Background:**

The clinical high-risk for psychosis (CHR-P) paradigm was introduced to detect individuals at risk of developing psychosis and to establish preventive strategies. While current prediction of outcomes in the CHR-P state is based mostly on the clinical assessment of presenting features, several emerging biomarkers have been investigated in an attempt to stratify CHR-P individuals according to their individual trajectories and refine the diagnostic process. However, heterogeneity across subgroups is a key challenge that has limited the impact of the CHR-P prediction strategies, as the clinical validity of the current research is limited by a lack of external validation across sites and modalities. Despite these challenges, electroencephalography (EEG) biomarkers have been studied in this field and evidence suggests that EEG used in combination with clinical assessments may be a key measure for improving diagnostic and prognostic accuracy in the CHR-P state. The PSYSCAN EEG study is an international, multi-site, multimodal longitudinal project that aims to advance knowledge in this field.

**Methods:**

Participants at 6 international sites take part in an EEG protocol including EEG recording, cognitive and clinical assessments. CHR-P participants will be followed up after 2 years and subcategorised depending on their illness progression regarding transition to psychosis. Differences will be sought between CHR-P individuals and healthy controls and between CHR-P individuals who transition and those who do not transition to psychosis using data driven computational analyses.

**Discussion:**

This protocol addresses the challenges faced by previous studies of this kind to enable valid identification of predictive EEG biomarkers which will be combined with other biomarkers across sites to develop a prognostic tool in CHR-P. The PSYSCAN EEG study aims to pave the way for incorporating EEG biomarkers in the assessment of CHR-P individuals, to refine the diagnostic process and help to stratify CHR-P subjects according to risk of transition. This may improve our understanding of the CHR-P state and therefore aid the development of more personalized treatment strategies.

## Introduction

Clinical high-risk for psychosis (CHR-P) designation (1) has the goal of altering the course of psychotic disorders through indicated prevention strategies (2). CHR-P individuals accumulate several risk factors for psychotic disorders (2, 3) and the majority display attenuated psychotic symptoms (4), in addition to a decline in functioning (5) and help-seeking behavior (6). Because of these features, their risk of developing a psychotic disorder within 2 years is 22% (7), although this rate varies across different CHR-P subgroups (8–10). Despite these achievements, some key challenges have limited the impact of the CHR-P prediction strategy. Firstly, we cannot detect all individuals who will later develop psychosis (11) or formulate a prediction of their outcomes beyond group-level prognostication (12). Secondly, current prediction of outcomes is generally based on the clinical assessment of presenting features and may not be reliable (13). Thirdly, no common, upstream neurobiological trigger for psychosis is currently known (14). To overcome these barriers, over the recent decade, extensive research has investigated the underlying neurobiological abnormalities in CHR-P individuals (7, 15).

Several emerging neuroimaging biomarkers have been investigated to stratify CHR-P individuals according to their individual trajectories (16) and may therefore be suitable as diagnostic markers to forecast the probability of a certain condition to be present and prognostic markers to forecast the probability of a certain outcome to occur (15).

Although electrophysiological (17), neuro-anatomical (18, 19), and blood markers (20) have been considered as predictors in this group, their clinical validity is limited by a lack of external validation across sites and modalities, despite attempts to do so. Due to this lack of validation, their use has been limited in precision psychiatry compared to other predictors such as clinical or socio-demographic predictors (21). However, with the expansion of multi-site data collection, there is growing evidence for electroencephalography (EEG) predicting clinical outcomes in CHR-P samples (22). Indeed, EEG used *in combination* with clinical assessments (23) has been investigated with highly promising results (24).

Reduced gamma band responses, at around 40 Hz, are a robust EEG alteration in established schizophrenia (25). A proposed mechanism links dysfunction in high frequency neural oscillations (>20 Hz) with psychotic symptoms (26) and cognitive impairments in schizophrenia (27), as gamma oscillations are fundamental for cognitive function (28) *via* cortico-cortical communications (29). According to previous research, gamma band activity may also have predictive properties to inform individual risk assessment in CHR-P subjects (30). In addition to such high frequency oscillations, theta (4–7 Hz) and delta (1–4 Hz) are also promising EEG biomarkers for CHR-P individuals, since resting state theta and delta are increased in chronic psychosis (31). Increased frontal theta has been reported in CHR-P individuals (32), and theta and delta alterations may also have predictive properties in this population (33, 34).

EEG is an investigational technique that has excellent temporal resolution, directly measures electrical neuronal activity and is relatively inexpensive to implement. Additionally, with the inclusion of an automated measurement pipeline for analysis purposes, EEG is easy to run without major training and is therefore an ideal modality for use in clinical practice.

PSYSCAN is an international, multi-site, multimodal and longitudinal project which will use machine learning techniques to analyze imaging, clinical, cognitive, and biological data to facilitate the prediction of psychosis onset and outcome (35). To test the potential utility of gamma band oscillations as diagnostic and prognostic biomarkers in CHR-P individuals, while at the same time controlling for the current limitations of research, large scale studies including CHR-P and healthy controls are required and this is one of the PSYSCAN project's core objectives. The current manuscript describes the PSYSCAN EEG protocol, which investigates oscillatory and event related activity in CHR-P individuals. It is hoped that this study will pave the way for incorporating EEG biomarkers in the assessment of CHR-P individuals, to refine the diagnosis process and improve the prognosis of mental health outcomes.

## Methods

### Design

The study design is longitudinal and includes two independent groups consisting of CHR-P individuals and healthy controls. CHR-P participants will be followed up after 2 years to assess illness outcome and be further subcategorised. Assuming that ~22% of the CHR-P group will develop psychosis over 2 years (7), this will yield subgroups who do or do not develop psychosis.

**Figure 1:**
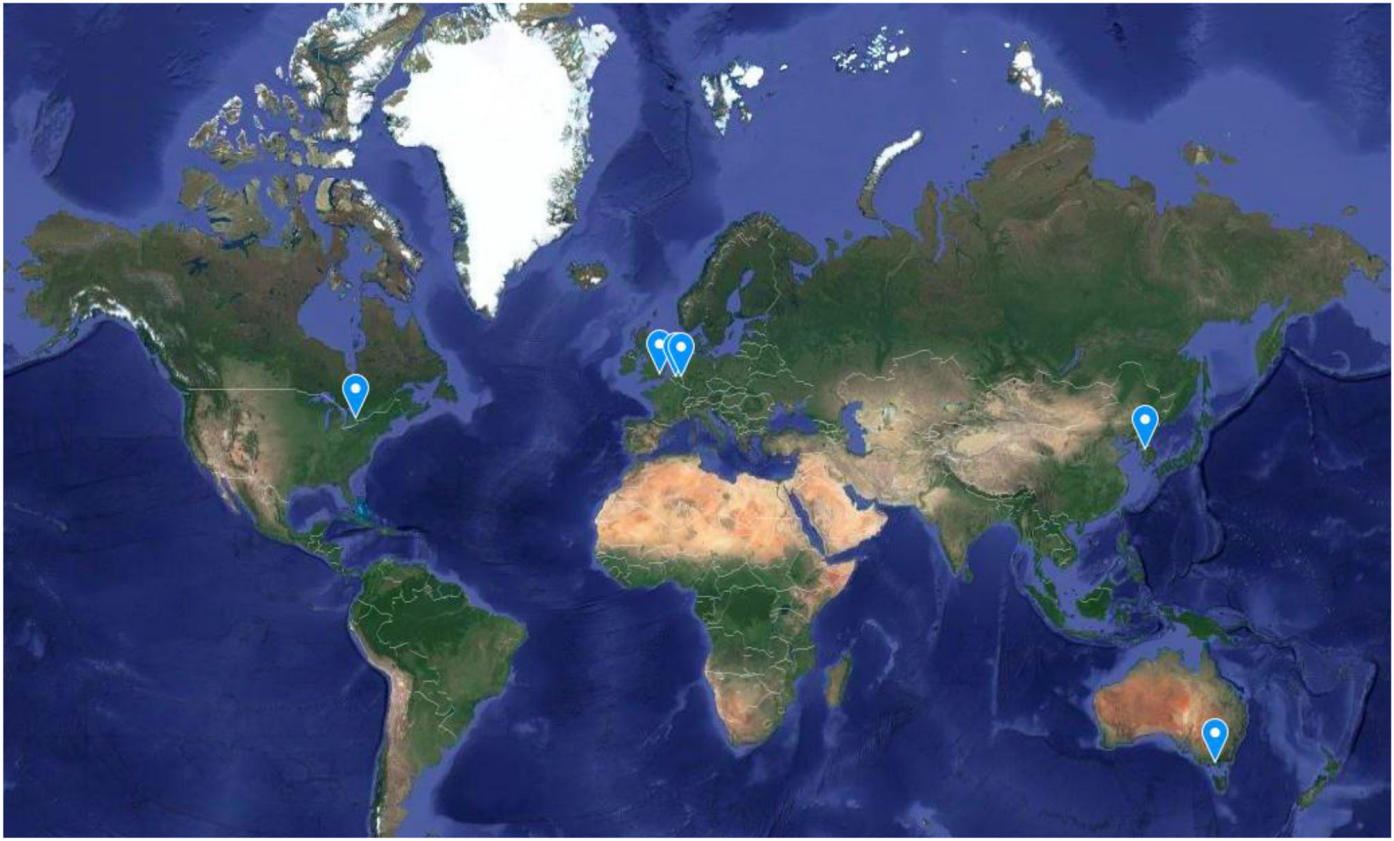
Participating sites.

The participating sites are: London, Amsterdam, Maastricht, Melbourne, Seoul, and Toronto (Figure 1). All participants will undergo EEG data acquisition in addition to PSYSCAN-related measures such as neuroimaging, biological, cognitive, and clinical assessments of symptomatology [see (35) for PSYSCAN-related procedures]. Differences will be sought between CHR-P individuals and healthy controls and between CHR-P individuals who transition and those who do not transition to psychosis using data driven computational analyses.

### EEG Data

#### EEG Data Acquisition

The different sites have a range of EEG amplifiers and recording facilities. At the London site, a Compumedics Neuroscan SYNAMPS2 amplifier is used and a Brain Products EEG cap. EEG recording take place in an electrically shielded EEG laboratory at the NIHR and Wellcome Trust funded King's Clinical Research Facility, King's College Hospital. The minimum sampling rate required for all sites in the study is 500 Hz (0.05–100 Hz filter settings). However, at sites with a suitable EEG amplifier, a fast sampling rate of 5 kHz (with 0.05 Hz−1 kHz filter settings) is used to allow for advanced artifact correction for gamma band analyses [see: (36)]. The left mastoid (M1) serves as the online reference channel, a right mastoid (M2) and the nose electrode is included for offline re-referencing, and the ground is at AFz. Electrodes are also placed outer to the eyes to capture vertical and horizontal electrooculograms (VEOU, VEOL, HEOL, and HEOR). All sites record a minimum of 32 EEG channels according to the 10–20 system. However, sites with the technological capability utilize extra electrodes from the 10–10 system and include additional inferior temporal (FT9 and FT10 and Left and Right Cheek), nasion, and cerebellar electrodes (PO09 and PO010) as well as an externally placed electrode to record the powerline noise.

#### Computerized Tasks Included in the PSYSCAN EEG Study

**Figure 2:**
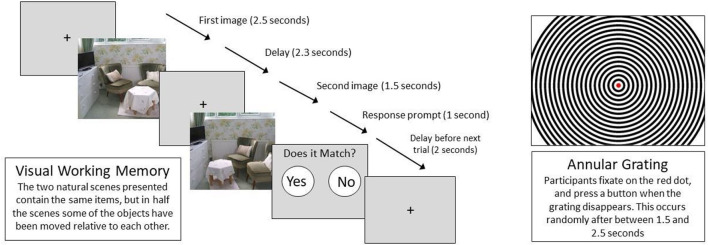
Visual paradigms.

- **Auditory Steady State Response (ASSR):** The ASSR 40 Hz condition has previously reported a diminished induced gamma band amplitude and is reported to predict individual risk assessment in CHR-P subjects (30). Subjects listen passively to a series of 20, 36, 40, and 44 Hz click trains through a pair of earphones. The computer screen is switched off to avoid unnecessary screen refresh artifacts and participants are instructed to close their eyes for the duration of the task. The click trains are 750 ms long, with 600 ms inter-stimulus intervals and there are 72 click trains of each frequency.
- **Pitch Deviant Auditory Oddball With Eyes Closed:** The P300, recorded in the Auditory Oddball paradigm, is a robust event-related potential (ERP) marker often used in EEG research and known to be reduced in CHR-P (37) as well as in first episode psychosis (38). However, the current study aims to assess the evoked gamma and theta spectral components in this paradigm as a common methodological issue may lead to inaccurate detection of gamma activity. It is reported that gamma activity may be generated by extra ocular muscles at the back of the eyes associated with saccades (39) rather than with cognitive processes. To avoid this confound, participants are instructed to keep their eyes closed. In addition, the computer screen is turned off for this task to reduce unnecessary screen refresh artifacts. In the current paradigm, participants are required to press a button in response to a target tone (1,000 Hz, 50 ms duration) in a series of standard tones (500 Hz, 50 ms duration). There are 55 target tones, with a 20% probability of a target tone occurring. The time between tones is randomly jittered between 1,066 and 1,288 ms.
- **Resting State:** Participants are instructed to “relax but try not to fall asleep”. Instructions through the in-ear earphones indicate 30 s periods of eyes open and eyes closed. An additional 5 min of eyes closed is recorded at the end of this period to best ensure a minimum of 2 min clean eyes closed data. The computer monitor is switched off. If a participant is observed to be falling asleep in any task, they are woken up and actions are recorded on the session run sheet.
- **Visual Working Memory (VWM):** This paradigm is intended to probe memory related theta and gamma oscillations. Theta is impaired in a visual delayed matching to sample memory task in people with schizophrenia (40), “Configural-relational” memory depends on hippocampal function and theta oscillations (41, 42). Theta synchronizes between the hippocampus and the neocortex in humans (43) and gamma-theta synchronization occurs during visual memory (44), so neocortical theta and gamma should be detectable in the scalp EEG. In the current visual working memory task, participants are presented with two natural scenes with a 2.3 s delay between them (see Figure 2). Both scenes are made up of identical items but in some pairs of images, one or more of the objects are in a different location in the second scene. The participant must indicate if the two pictures match by a button press for “yes” or “no”. Where necessary, the “Yes/No” in the response prompt has been translated into the language suitable for that site. There are 50 image pairs used in each session (25 matched and 25 mismatched) randomly selected from 78 possible images.
- **Visual Annular Grating:** A black and white circular grating appears on the screen with a central, small red dot (see Figure 2). Participants are instructed to fixate on the dot and to press a button as soon as possible when the grating disappears. This task is known to produce an occipital gamma signal (45) which we will investigate. There are 50 stimuli, presented every 5 s. The time that the gratings are visible is randomly varied between 1.5 and 2.5 s.

#### EEG Analysis

##### High Frequency Analyses (>20 Hz)—Matlab

EEG pre-processing and analysis will be completed using Matlab for gamma band investigations. Powerline, eye and muscle artifacts will be removed using a gamma artifact removal algorithm developed by our researchers to remove artifacts without excluding EEG high frequency oscillatory activity [see (36)]. Fast Fourier Transform (FFT) will then be performed to convert the EEG signal to time-frequency domain for extraction of the amplitude of gamma band activity. The time-locked, gamma response, as in the ASSR, can be determined by first averaging across trials, before performing the FFT. This averaging reduces the effect of artifacts, allowing data collected at a lower sampling rate to be used. After decimating to the same sampling rate, data from all sites will be subject to identical processing pipelines for this time-locked gamma response, prior to group averaging. However, the technical requirements for artifact correction of the exploratory, resting and non-time-locked (induced) high frequency analyses, may mean that data from some sites might need to be excluded from such additional high frequency analyses. Whilst the gamma response in the ASSR is the primary outcome measure for this study, the EEG recorded in the Annular Grating, VWM, Auditory Oddball and Resting paradigms will also undergo analyses of the frequencies above 20 Hz. Detailed analysis methods, including relevant software pipelines will be reported with results, in future publications.

##### Low Frequency Analysis and ERPs–Brain Vision Analyzer2

The P300, Resting State and VWM task will be subject to confirmatory analyses of ERPs (46), peak alpha, frontal theta and delta (34) and theta band activity (33), respectively, using Brain Vision Analyzer2 (47) in the large, global sample we will have available to us. The inclusion of these analyses is crucial as they may have contributary predictive properties as previously reported. Further spectral analyses will additionally be conducted on the data acquired on the VWM paradigm and the resting state to assess synchrony across brain regions and frequency bands.

### Informed Consent, Data Acquisition, and Visits

During the screening visit, the study is explained to potential participants both verbally and in written form by a Participant Information Sheet (PIS) where the aims, methods, anticipated benefits, and potential hazards are described. Participants are given ample time to consider whether they wish to take part and to have any questions they may have answered. It is made clear that participation is voluntary, that all clinical and EEG data collected will be anonymized, and that it is the participants' right to withdrawal from the study at any time without giving a reason. Informed written consent is obtained for those individuals that meet inclusion criteria and wish to participate in the study. Both CHR-P individuals and controls are evaluated at baseline, completing a clinical assessment, a cognitive assessment, and an EEG assessment and resting state magnetic resonance imaging (rsMRI) assessment are carried out (see Table 1). All the data are acquired in accordance with the PSYCAN protocol [see: (35)]. Participants receive a small amount of financial compensation for their time and to cover any travel costs they may incur.

**Table 1:** Visits and study procedures in CHR-P individuals and HC.

	**Screening visit**	**Baseline visit**	**24 months visit^a^**
Participant information and informed consent	X		
Clinical assessment		X	X
Cognitive assessment (*Hopkins Verbal Learning Test Revised*)		X	X
EEG assessment		X	X

a *Timing is relative to the baseline visit ±1 month*.

### Data Analysis

#### Sample Size

The study is powered on the primary hypothesis that there are gamma band differences between CHR-P and HC groups. Given that there are no large-scale studies addressing gamma band alterations in CHR-P samples we have used the effect size from a meta-analysis conducted in patients with a first episode of psychosis (25). Power calculations using G^*^Power reveal that, using the meta-analytical pooled effect size of 0.6, the sample size required (two-tailed alpha value of 0.05, power of 80%, HC:CHR-P allocation ratio of 0.4) to detect this effect is 79 CHR-P participants and 31 HC. Our sample size will exceed these numbers to ensure that we detect group differences in EEG measures if present in participants. However, the study is primarily powered to detect differences between CHR-P individuals and HC. As such, the study is not powered to differentiate those who will develop psychosis or not.

#### Statistical Analysis

Differences between HC and CHR-P subgroups will be calculated using *t*-test. To investigate associations of gamma band oscillations with structural and functional alterations, correlation analyses will be applied to test relationships between gamma amplitude, blood oxygen level dependent (BOLD) response acquired during the neuroimaging protocol and clinical presentation as assessed using the Comprehensive Assessment of At-Risk Mental States (CAARMS). Finally, all measures will be subject to data driven computational analyses to develop and validate predictors of outcome in conjunction with other data modalities from the PSYSCAN project [For PSYSCAN measures, standardization methods and computational analysis aims see (35)].

### Participants

CHR-P participants will be recruited from CHR-P services at each site (see Section EEG Data for details). All participants taking part in this EEG study will have already been recruited and enrolled for the PSYSCAN study [see (35)]. Healthy controls are recruited from the local population through advertisements in the local areas local to each site. PSYSCAN data is being collected as part of a multisite project involving numerous sites with established experience in recruiting the CHR-P patient population to increase sample size, variability, and representation. As such it is unlikely that the recruited population will substantially differ from that typically assessed in these sites. However, while efforts were made to match HCs on age, years of education, race, and sex where possible, the study is limited to those who are referred to participating clinics and meet the CHR threshold criteria. Therefore, PSYSCAN will address the core sociodemographic factors that account for risk enrichment in this population, as identified by recent studies (2), including age, gender, ethnicity, and socioeconomic status.

#### Inclusion Criteria

- CHR-P individuals fulfilling one or more of the following criteria (i-iii according to CAARMS 12/2006) (48) and iv according to the Schizophrenia Proneness Instrument—Adult version (SPI-A) (49):
  (i) Attenuated Psychotic Symptoms (APS): characterized by attenuated positive symptoms (ideas of reference, odd beliefs/magical thinking, perceptual disturbance, digressive speech, odd behavior/appearance).
  (ii) Brief intermittent psychotic symptoms (BLIPS): characterized by full-blown psychotic symptoms which last less than one week and resolve spontaneously.
  (iii) Genetic Risk and Deterioration (GRD): characterized by a first degree relative of an individual with a psychotic disorder and/or a schizotypal personality disorder, plus a marked reduction in Global Assessment of Function score.
  (iv) Basic Symptoms (BS): subtle, subclinical self-experienced disturbances in drive, stress tolerance, affect, thinking, speech, perception and motor action.
- Aged 16–40 years old.
- All participants must speak and understand English to a standard to provide informed consent and follow EEG paradigm instructions.
- Healthy controls must have no personal or family history of psychiatric disorders.

#### Exclusion Criteria

- Present or past diagnosis of DSM psychotic disorder.
- Evidence of neurological conditions, major medical illness or head injury.
- Severe skin reactions to cosmetics.

### Outcomes

Primary outcome:

- Differentiate CHR-P individuals and HC based on gamma band EEG data.

Secondary outcomes:

- Develop and validate predictors of outcome including psychosis onset in conjunction with the PSYSCAN project.
- Refine predictive models using EEG data and other biomarkers evaluated in the PSYSCAN project.
- Evaluate the influence of clinical, cognitive, neuroimaging, and biological biomarker data collected for the PSYSCAN project on CHR-P outcomes.

### Data Management

#### Methodological Considerations in Multi-Site EEG Studies

Multi-site studies provide the opportunity to collect large samples of data from a broad population. However, the involvement of multiple sites also introduces an increase in methodical challenges in controlling for site differences. At the data collection, storage, analysis and interpretation levels, important standardization measures need to be taken [for overview see: (50)]. For the current study, differing parameters that can be altered in the pre-processing stages have been employed for future directions. For example, while some sites have the resources for collecting data at a sampling rate of 5,000 Hz, for the main inter-group analyses, sampling rates will be decimated to the limits of the lowest sampling rates available at any site. The same procedure must be applied to the limitations of all parameters, e.g., channel selection. However, the higher sampling rate and extra channels will allow further analyses to be carried out on data from a sub-set of the sites.

#### Traveling Heads

While every effort has been made to reduce between-site effects through the methodology and standard operating procedures (SOP) protocol, variability may still arise from differing make or model of acquisition or presentation equipment and software. To measure these differences, six healthy subjects—or “Traveling Heads”—underwent two EEG sessions on consecutive days at each of the six participating sites. This approach, adopted in previous multi-site studies, will allow for assessment of heterogeneity within- and between-laboratory settings such as acquisition and presentation equipment, and room condition (e.g., temperature, lighting, and electromagnetic noise). Determinants and degrees of variance can then be assessed for their effects on data and applied to *post-hoc* calibration methods for attenuation.

#### Data Security

Data Security Privacy laws and regulations are adhered to for all procedures undertaken during this study. The collection and processing of participants' personal information is limited to the details as defined and approved in the ethics application. All data and personal information collected from participants during the investigation is treated with the strictest confidentiality. Once recruited and consented to the project, all participants are allocated a participant ID number which is attached to all research documentation. All documentation collected, which would allow the identification of personal data, is stored in a secure location at King's College London's Institute of Psychiatry, Psychology and Neuroscience in a locked cabinet and only accessible by the researcher and the CI. All clinical and EEG data is anonymized and stored under password protection on the King's College London's Institute of Psychiatry, Psychology and Neuroscience on our secure, encrypted server. Research data will be stored for a minimum of 5 years following the completion of the study and for follow-up purposes. Anonymised clinical and EEG data from the Amsterdam, Maastricht, Melbourne, Seoul, and Toronto are transferred to the London site also *via* our secure, password-protected, encrypted server.

### Ethics and Regulatory Approval

This study was reviewed and given a favorable opinion by the National Research Ethics Service (NRES) London—Fulham Research Ethics Committee (Ref: 16/LO/1829), in accordance with the Helsinki Declaration of 1975 and 2008 amendment and with the UNESCO Universal Declaration on human rights. The study is being conducted according to the principles of the Declaration of Helsinki (Amendment, 2008), and all applicable regulatory requirements.

### Acknowledgments of Financial Support

The PSYSCAN Project was supported by Grant Agreement No. 241909 under the European Union's Seventh Framework Program.

## Discussion

The current manuscript presents the protocol for an international, multi-modal and longitudinal study that seeks to identify predictors of outcome in CHR-P individuals; running in conjunction with the PSYSCAN study. The primary investigative technique used is EEG and the primary variable of interest is the spectral response in the gamma band. Further, investigatory spectral analyses will also be applied to assess amplitude and synchrony across brain regions and frequency bands during rest and cognitive tasks. Participants will be followed up over 2 years and potential transition to psychosis will be evaluated. The successful identification of predictors of outcome may have the potential to aid development of an EEG assessment that would refine individual risk prediction in the CHR-P state along with other biomarkers. To our knowledge, this is the first international, multi-modal, multi-site, longitudinal study of this scale investigating the gamma band response as a predictor of outcome in the CHR-P state.

We believe that the protocol described here address some of the challenges faced by previous studies of this kind, including the limitation of precision psychiatry to clinical or socio-demographic predictors only to predict poor outcomes (21). It will therefore enable us to identify predictive biomarkers which will inform clinical practice. An achievable goal would be generating a stepwise, multi-level assessment analogous to the reliable, sequential, diagnostic testing employed for other medical vulnerabilities (e.g., myocardial infarction) as this method may provide the greatest refinement of prognosis accuracy (15). Therefore, the multi-modal nature of our study has been designed to allow for the stepwise consideration of variables with a goal of generating a valid risk assessment model for implementation in the clinical environment. A potential limitation of the current study protocol is that CHR-P subjects are not compared to subjects with non-psychotic psychiatric disorders or subclinical symptoms, which can limit interpretation of results (51). However, PSYSCAN does not include a group of help-seeking controls because the primary aim is to develop diagnostic and prognostic biomarkers. As such the main control group of interest consists of healthy controls. This would mean that PSYSCAN is not suited to address the heterogeneous risk enrichment that is observed in help-seeking samples accessing specialized services for psychosis prevention. Our group has elaborated on these issues in precedent publications (3, 7, 52, 53).

## Study Status

The study status is ongoing. Recruitment commenced in January 2017 and ethical approval to collect data ended in September 2021. Data collection was being conducted according to the principles of the Declaration of Helsinki (Amendment, 2008), and all applicable regulatory requirements. This study was reviewed and given a favorable opinion by the National Research Ethics Service (NRES) London—Fulham Research Ethics Committee (Ref: 16/LO/1829).

## Ethics Statement

The studies involving human participants were reviewed and approved by National Research Ethics Service (NRES) London—Fulham Research Ethics Committee (Ref: 16/LO/1829). Written informed consent from the participants' legal guardian/next of kin was not required to participate in this study in accordance with the national legislation and the institutional requirements.

## PSYSCAN Consortium

London—Study Co-ordination: Philip McGuire^1^, Stefania Tognin^1^, Paolo Fusar-Poli^1^, Matthew Kempton^1^, Gemma Modinos^1^, Kate Merritt^1^, Alexis E. Cullen^1^, Andrea Mechelli^1^; London—UHR + FEP recruitment: Paola Dazzan^2^, George Gifford^1^, Natalia Petros^1^, Mathilde Antoniades^1^, Andrea De Micheli^1^, Sandra Vieira^1^, Tom Spencer^1^; Utrecht—Study Co-ordination and Recruitment: Rene Kahn^3, 4^, Arija Maat^3^, Erika van Hell^3^, Inge Winter^3^; Amsterdam: Lieuwe de Haan^5^, Frederike Schirmbeck^5^; Cantabria: Benedicto Crespo-Facorro^6,7^, Diana Tordesillas-Gutierrez^6,7^, Esther Setien-Suero^6,7^, Rosa Ayesa-Arriola^6,7^, Paula Suarez-Pinilla^6,7^, Victor Ortiz Garcia-de la foz^6,7^; Copenhagen: Birte Glenthøj^8,9^, Mikkel Erlang Sørensen^8^, Bjørn H. Ebdrup^8,9^, Karen Tangmose^8,9^, Helle Schæbel^8^, Egill Rostrup^8,10^; Heidelberg: Oliver Gruber^11^, Anja Richter^1,11^, Bernd Krämer^11^; Maastricht: Therese van Amelsvoort^12^, Bea Campforts^12^, Machteld Marcelis^12, 13^, Claudia Vingerhoets^12^; Madrid: Celso Arango^14^, Covandonga M. Díaz-Caneja^14^, Miriam Ayora^14^, Joost Janssen^14^, Roberto Rodríguez-Jiménez^15^, Marina Díaz-Marsá^16^; Marburg: Tilo Kircher^17^, Irina Falkenberg^17^, Florian Bitsch^17^, Jens Sommer^17^; Melbourne: Barnaby Nelson^18,19^, Patrick McGorry^18,19^, Paul Amminger^18,19^, Meredith McHugh^18,19^, Suzie Lavoie^18^, Jessica Spark^18^, Rebekah Street^18^; Naples: Silvana Galderisi^20^, Armida Mucci^20^, Paola Bucci^20^, Giuseppe Piegari^20^, Daria Pietrafesa^20^, Luigi Giuliani^20^, Rodrigo Bressan^21^; São Paulo: André Zugman^21^, Ary Gadelha^21^, Graccielle Rodrigues da Cunha^21^; Seoul: Jun Soo Kwon^22^, Kang Ik Kevin Cho^22^, Tae Young Lee^22^, Minah Kim^22^, Sun-Young Moon^22^, Silvia Kyungjin Lho^22^; Tel HaShomer: Mark Weiser^23^; Toronto Data Acquisition, Montreal Coordination: Romina Mizrahi^24,25,26^, Michael Kiang^24,25,26^, Cory Gerritsen^25,27^, Margaret Maheandiran^25^, Sarah Ahmed^24,25^, Ivana Prce^25^, Jenny Lepock^24,26^; Vienna: Gabriele Sachs^28^, Matthäus Willeit^28^, Marzena Lenczowski^28^, Ullrich Sauerzopf^28^, Ana Weidenauer^28^, Julia Furtner-Srajer^29^; Zurich: Matthias Kirschner^30,31^, Anke Maatz^30^, Achim Burrer^30^, Philipp Stämpfli^30^, Naemi Huber^30^, Wolfram Kawohl (UHR)^32^

^1^Department of Psychosis Studies, Institute of Psychiatry, Psychology and Neuroscience, King's College London, London, United Kingdom

^2^Department of Psychological Medicine, Institute of Psychiatry, Psychology and Neuroscience, King's College London, London, United Kingdom

^3^University Medical Center, Division of Neurosciences, Department of Psychiatry, Utrecht, Netherlands

^4^Department of Psychiatry and Behavioral Health System, Icahn School of Medicine at Mount Sinai, New York, NY, United States

^5^Department of Psychiatry, Early Psychosis, Amsterdam UMC, University of Amsterdam, Amsterdam, Netherlands

^6^Department of Psychiatry, Marqués de Valdecilla University Hospital, IDIVAL, School of Medicine, University of Cantabria, Santander, Spain

^7^Hospital Universitario Virgen del Rocio, Universidad de Sevilla, IBiS, CIBERSAM, Sevilla, Spain

^8^Centre for Neuropsychiatric Schizophrenia Research (CNSR), Centre for Clinical Intervention and Neuropsychiatric Schizophrenia Research (CINS), Mental Health Centre Glostrup, University of Copenhagen, Glostrup, Denmark

^9^Department of Clinical Medicine, Faculty of Health and Medical Sciences, University of Copenhagen, Copenhagen, Denmark

^10^Functional Imaging Unit (FIUNIT), Rigshospitalet Glostrup, University of Copenhagen, Glostrup, Denmark

^11^Section for Experimental Psychopathology and Neuroimaging, Department of General Psychiatry, Heidelberg University, Heidelberg, Germany

^12^Department of Psychiatry and Neuropsychology, Maastricht University, Maastricht, Netherlands

^13^GGZE Mental Health Care, Eindhoven, Netherlands

^14^Servicio de Psiquiatría del Niño y del Adolescente, Hospital General Universitario Gregorio Marañon, Universidad Complutense Madrid, Spain; Centro de Investigación Biomédica en Red de Salud Mental, Madrid, Spain

^15^Departmento de Psiquiatría, Instituto de Investigación Sanitaria Hospital 12 de Octubre (imas12), Madrid, Spain; CIBERSAM (Biomedical Research Networking Centre in Mental Health), Spain

^16^Hospital Clínico de San Carlos, Universidad Complutense, Centro de Investigación Biomédica en Red de Salud Mental (CIBERSAM), Madrid, España.

^17^Department of Psychiatry, University of Marburg, Rudolf-Bultmann-Straße 8, Marburg, Germany

^18^Orygen, Parkville, Melbourne, VIC, Australia

^19^Centre for Youth Mental Health, The University of Melbourne, Parkville, VIC, Australia

^20^Department of Psychiatry, University of Campania Luigi Vanvitelli, Largo Madonna delle Grazie, Naples, Italy

^21^Department of Psychiatry, Interdisciplinary Lab for Clinical Neurosciences (LiNC), Universidade Federal de São Paulo (UNIFESP), São Paulo, Brazil

^22^Department of Psychiatry, Seoul National University College of Medicine, Seoul, South Korea

^23^Department of Psychiatry, Sheba Medical Center, Tel Hashomer, Israel; Sackler School of Medicine, Tel Aviv University, Tel Aviv, Israel

^24^Institute of Medical Science, University of Toronto, Toronto, ON, Canada

^25^Centre for Addiction and Mental Health, Toronto, ON, Canada

^26^Department of Psychiatry, McGill University, Montreal, QC, Canada

^27^Department of Psychology, University of Toronto, Toronto, ON, Canada

^28^Department of Psychiatry and Psychotherapy, Vienna, Austria

^29^Department of Biomedical Imaging and Image-Guided Therapy, Medical University of Vienna, Vienna, Austria

^30^Department of Biomedical Imaging and Image-guided Therapy, Medical University of Vienna, Vienna, Austria

^31^Department of Psychiatry, Psychotherapy and Psychosomatics, Psychiatric Hospital, University of Zurich, Zurich, Switzerland

^32^Department for Psychiatry and Psychotherapy, Psychiatric Services Aargau, Brugg, Switzerland

## Author Contributions

PM gained funding. PM, PF-P, and JN designed the study. SK is acquiring the data. SK and JN will analyze the data. SK and GS drafted this manuscript which was edited by PF-P and JN. All authors read and approved the final manuscript.

## Funding

The PSYSCAN Project was supported by Grant Agreement No. 241909 under the European Union's Seventh Framework Program. It has been funded by the National Institute of Mental Health in the USA and the European Commission. BN was supported by an NHMRC Senior Research Fellowship (1137687).

## Conflict of Interest

TA was employed by GGZE Mental Health Care. BN was employed by Orygen, The National Centre of Excellence in Youth Mental Health. The remaining authors declare that the research was conducted in the absence of any commercial or financial relationships that could be construed as a potential conflict of interest.

## Publisher's Note

All claims expressed in this article are solely those of the authors and do not necessarily represent those of their affiliated organizations, or those of the publisher, the editors and the reviewers. Any product that may be evaluated in this article, or claim that may be made by its manufacturer, is not guaranteed or endorsed by the publisher.
